# Effect of Oxygen Concentration and Tantalum Addition on the Formation of High Temperature Bismuth Oxide Phase by Mechanochemical Reaction

**DOI:** 10.3390/ma12121947

**Published:** 2019-06-17

**Authors:** Hsiu-Na Lin, May-Show Chen, Yu-Hsueh Chang, Pee-Yew Lee, Chung-Kwei Lin

**Affiliations:** 1 Research Center of Digital Oral Science and Technology, College of Oral Medicine, Taipei Medical University, Taipei 110, Taiwan; tiffanylin1214@gmail.com (H.-N.L.); mayshowc@hotmail.com (M.-S.C.); 2 Department of Dentistry, Chang Gung Memorial Hospital, Taipei 105, Taiwan; 3 Division of Prosthodontics, Department of Dentistry, Taipei Medical University Hospital, Taipei 110, Taiwan; 4 School of Dentistry, College of Oral Medicine, Taipei Medical University, Taipei 110, Taiwan; 5 Institute of Materials Engineering, National Taiwan Ocean University, Keelung 202, Taiwan; 10255002@ntou.edu.tw; 6 School of Dental Technology, College of Oral Medicine, Taipei Medical University, Taipei, 110, Taiwan

**Keywords:** bismuth oxide, phase transformation, high temperature metastable phase, mechanical milling, mechanochemical reaction

## Abstract

High-temperature face-centered cubic bismuth oxide phase is a material of great interest given its unique properties. In the present study, α-Bi_2_O_3_ and tantalum powders were used as the starting powders for the formation of high-temperature bismuth oxide phase via mechanochemical synthesis by high energy ball milling. (Bi_2_O_3_)_80_(Ta)_20_ and (Bi_2_O_3_)_95_(Ta)_5_ in weight concentrations were milled in either an oxygen-free argon-filled glove box environment or an ambient atmosphere to investigate the effects of oxygen concentration and tantalum addition. The as-milled powders were examined using X-ray diffraction, scanning electron microscopy with energy-dispersive spectroscopy, and differential scanning calorimetry to reveal the structural evolution. The experimental results showed that for (Bi_2_O_3_)_95_(Ta)_5_ powder mixtures milled within the glove box, tantalum gradually reacted with the α-Bi_2_O_3_ phase and formed a β-Bi_7.8_Ta_0.2_O_12.2_ phase. For (Bi_2_O_3_)_80_(Ta)_20_ milled under the same conditions, Ta and α-Bi_2_O_3_ mechanochemically reacted to form δ-Bi_3_TaO_7_ and bismuth after 10 min of high energy ball milling, whereas milling (Bi_2_O_3_)_80_(Ta)_20_ under the ambient atmosphere with a much higher oxygen concentration accelerated the mechanochemical reaction to less than five minutes of milling and resulted in the formation of high-temperature δ-Bi_3_TaO_7_ phase.

## 1. Introduction

Bismuth oxide is attracting increasing R&D interest due to its applications as a dental radiopacifying material [1,2,3] and an ionic conductor in solid oxide fuel cells [4,5,6,7,8]. Bismuth oxide exhibits four different crystal structures: α-, β-, γ-, and δ-phases. Monoclinic α-Bi_2_O_3_ phase is in a stable phase at room temperature but transforms into an fcc δ-phase at 729 °C, and melts into a liquid phase at 825 °C. Tetragonal β- and bcc γ-phase are two possible metastable phases and appear at 650 and 639 °C, respectively, when cooling from high temperatures [9,10,11].

The δ-phase is one of the best oxide ionic conductors, but it is stable at temperatures ranging from 729 to 825 °C. Its fluorite-like structure induces 25% oxygen vacancies and cannot be stable at room temperature. Higher valence elements or smaller ionic radii are preferred to preserve the desired fluorite-like structure at room temperature. For example, TiO_2_ [12], ZrO_2_ [13], HfO_2_ [13], SnO_2_ [14], Nb_2_O_5_ [4,15], Ta_2_O_5_ [11,15,16,17], MoO_3_ [15], and WO_3_ [12,15] with four to six valence electrons have been used to attempt to stabilize the fluorite-like structure at room temperature. In most reported research, starting oxide powders (usually α-Bi_2_O_3_ with various amounts of the above-mentioned dopants) were mixed, ground, and annealed at desired temperatures with sufficient time to obtain the fcc δ-phase.

The high-energy ball milling process [18,19,20], an efficient method used to prepare materials that are difficult to synthesize using conventional melting and casting techniques, is an alternative method to obtain the fluorite-like structure. Through mechanical alloying of dissimilar powders and mechanical milling of a single starting powder, nonequilibrium phases, which include amorphous materials, extended solid solutions, intermetallic compounds, metastable crystalline materials, nanocrystalline powders, and quasicrystals, can be synthesized [18,20,21,22]. If the mechanical energy produced during ball milling is absorbed by the milled materials and triggers a chemical reaction, mechanochemical synthesis occurs [23,24,25]. For instance, iron nanoparticles have been prepared via mechanochemical synthesis using iron chloride and sodium as the starting powders [26].

A few high-temperature bismuth oxide phases have been prepared via the high-energy ball milling process. For example, a δ-Bi_2_O_3_ phase stabilized by hafnia and zirconia has been prepared using mechanochemical synthesis [13]. Castro and Palem [27] investigated the binary and ternary Bi_2_O_3_-Nb_2_O_5_-Ta_2_O_5_ oxide systems and fluorite-like Bi_3_NbO_7_, Bi_3_TaO_7_, and Bi_3_Nb_1-x_Ta_x_O_7_ phases were formed. Preparation of a metastable Bi_6_Ti_5_O_22_ phase was achieved by mechanochemical milling of Bi_2_O_3_, TiO_2_, and WO_3_ stoichiometric powder mixtures [12]. In addition, fully stabilized bcc γ-Bi_2_O_3_ phase was mechanosynthesized without any additive [28].

The aforementioned investigations involved milling α-Bi_2_O_3_ with one or two other high valency oxides. Adding these oxides is expected to stabilize the high-temperature δ-phase at room temperature and produce an excellent ionic conductor that can be used in solid oxide fuel cells. However, milling α-Bi_2_O_3_ and tantalum powder mixture has not been reported in the literature and may lead to unexpected mechanochemical reaction. Only Hasanpour et al. [29] reported that mechanochemical synthesis of Bi-Fe_3_O_4_ nanocomposite was achieved by milling Bi_2_O_3_ and Fe powder mixtures. In the present study, we prepared a high-temperature fluorite-like δ-Bi_2_O_3_ phase by milling α-Bi_2_O_3_ and tantalum powder mixtures. The effects of tantalum addition and oxygen concentration, as well as structural evolution of the high-temperature bismuth oxide phase formation during ball milling, were investigated.

## 2. Experimental Procedures

Commercially available α-Bi_2_O_3_ (99.9%) and tantalum (99.98%) powders were used as the starting powders. We canned 4 g of the desired (Bi_2_O_3_)_80_(Ta)_20_ or (Bi_2_O_3_)_95_(Ta)_5_ compositions in weight percentage and Cr steel balls (7 mm in diameter), which were selected to follow a ball-to-powder ratio of 5:1, into a SKH 9 high speed steel vial (40 mm in diameter and 50 mm in height). A SPEX 8000D shaker ball mill (Fisher Scientific, Ottawa, ON, Canada) was used for the high energy ball milling process, which was performed either under ambient atmosphere conditions or within an Ar-filled glove box, which had a total oxygen and water concentration of less than 100 ppm. The overall milling process lasted 3 h for (Bi_2_O_3_)_80_(Ta)_20_ and 10 h for (Bi_2_O_3_)_95_(Ta)_5_. The milling process was interrupted every 5 min for the first 30 min and interrupted every 30 min thereafter. Each interruption was followed by an equal length of time to cool the vial. An appropriate amount of the as-milled powder was extracted to examine the progress of the structural evolution.

The structural evolution during various milling stages were examined using X-ray diffraction (XRD), scanning electron microscopy (SEM), and differential scanning calorimetry (DSC). The XRD analysis was performed using a Bruker AXS GmbH-D2 PHASER diffractometer (Billerica, MA, USA) with monochromatic Cu-Kα radiation (30 kV and 10 mA). Phase percentages were determined by performing Rietveld fitting of the XRD patterns using XRD analysis software EVA (Bruker-AXS Diffrac EVA, Bruker, WI, USA). The cross-sectional views of as-milled powders were examined using a Nova NanoSEM 230 scanning electron microscope (FEI, Hillsboro, OR, USA) equipped with an EDAX Apollo X silicon drift detector EDS system (EDAX, Mahwah, NJ, USA) The DSC analysis was performed with a NETZSCH DSC 200 F3 differential scanning calorimeter (Gerätebau GmbH, Selb, Germany) where the sample was heated from room temperature to 300 °C in a purified argon atmosphere at a heating rate of 20 K/min.

## 3. Results and Discussion

**Figure 1:**
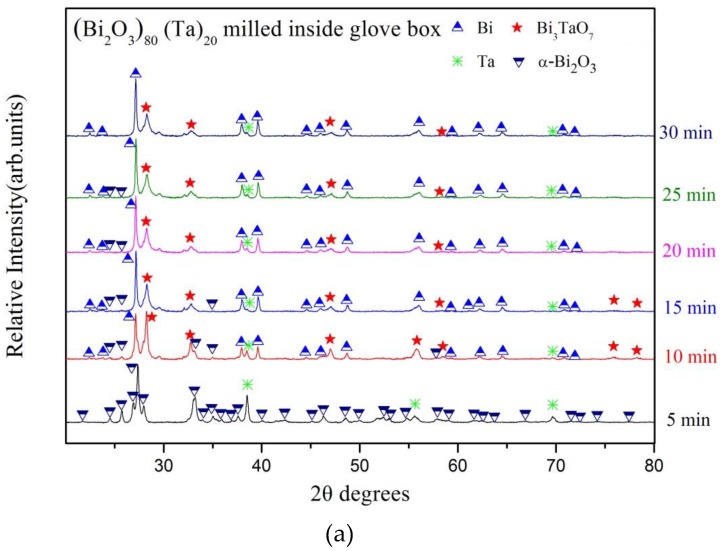

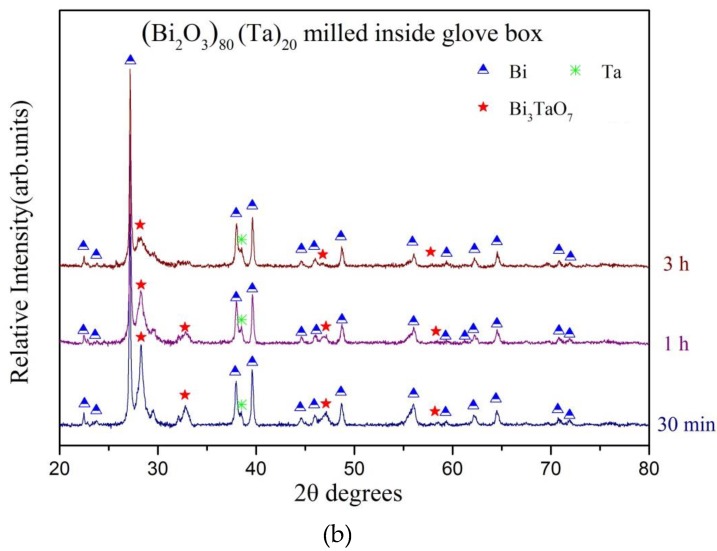
XRD patterns of (Bi_2_O_3_)_80_Ta_20_ as a function of milling time under Ar-filled glove box conditions: (**a**) early stages of milling and (**b**) long-term milling.

To understand the structural evolution during the ball milling process, we used XRD and cross-sectional observations using SEM. Figure 1 shows the XRD patterns as a function of milling time for the (Bi_2_O_3_)_80_(Ta)_20_ powder produced using high- energy ball milling within an argon-filled glove box environment. At the early stage of milling, up to the first 25 min, crystalline peaks of the α-Bi_2_O_3_ starting powder were observed (Figure 1a). The peaks’ intensities decreased with increasing milling time. A similar trend was observed for tantalum. The diffraction peaks of tantalum, however, could be observed throughout the whole three-hour milling process. In addition, three new crystalline phases, which were α-Bi_2_O_4_ (ICDD PDF card No. 50-0864), bismuth (ICDD PDF card No. 44-1246), and fluorite-like cubic δ-Bi_3_TaO_7_ (ICDD PDF card No. 044-0202), formed after only 10 min of ball milling. The peaks’ intensities of α-Bi_2_O_4_ did not show noticeable differences throughout the milling process (from 10 min to three hours). The relative amount of bismuth increased with increasing milling time until the end of the three hours of milling. The formation of α-Bi_2_O_4_, bismuth, and Bi_3_TaO_7_ suggests that tantalum reacts with α-Bi_2_O_3_ particles and the mechanochemical reaction occurs after only 10 min of high energy ball milling. This shows a behavior similar to one previously reported in literature, in which milling α-Bi_2_O_3_ and iron leads to the formation of Bi and Fe_3_O_4_ [29]. However, the relative peaks’ intensities of Bi_3_TaO_7_ did not exhibit significant differences at the early stage of milling (i.e., the first 10–30 min). Slight decreases in the peaks’ intensities and broadening of peaks was observed after long-term milling from 30 min to three hours (Figure 1b).

**Figure 2:**
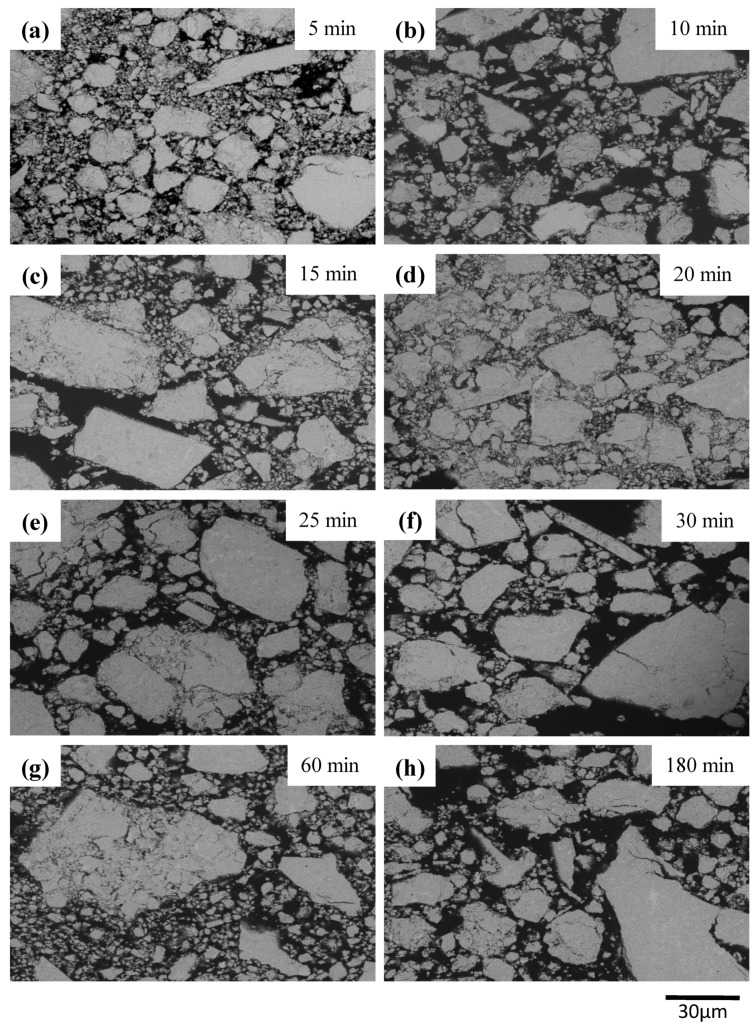
SEM images of (Bi_2_O_3_)_80_Ta_20_ as a function of milling time under Ar-filled glove box conditions: (**a**) 5, (**b**) 10, (**c**) 15, (**d**) 20, (**e**) 25, (**f**) 30, (**g**) 60, and (**h**) 180 min.

Figure 2 shows the cross-sectional SEM images of as-milled (Bi_2_O_3_)_80_(Ta)_20_ powder. Irregular grains with a wide particle size distribution were be observed at all milling stages starting from five minutes of milling (Figure 2a) to the end of three hours’ milling (Figure 2h). Typically, the high-energy ball milling process can be divided into three different types of milling mechanisms, depending on the characteristics of starting powders: both ductile, ductile/brittle, and both brittle [20]. During ball-powder-ball collision, powder particles undergo deformation and/or a fracture process. Ductile powders usually form a lamellar structure at the beginning and result in homogeneous equiaxed particles at the end of milling. For a ductile/brittle system, brittle particles may fracture and are trapped within ductile interfaces and finally form a metal matrix composite with a uniform distribution of embedded brittle particles, whereas brittle/brittle particles are expected to fracture continuously into small particles and sometimes mechanical alloying between starting particles may occur. The present milling system consisted of 20 wt % ductile Ta and 80 wt % brittle α-Bi_2_O_3_ particles. The densities of Ta and α-Bi_2_O_3_ were 16.69 and 8.9 g/cm^3^, respectively. The relative volume of ductile Ta is low and unable to trap all the fractured brittle Bi_2_O_3_ particles within the interfaces. This suggests that the powders were fractured and agglomerated repetitively throughout the milling process. No lamellar structure was observed.

**Figure 3:**
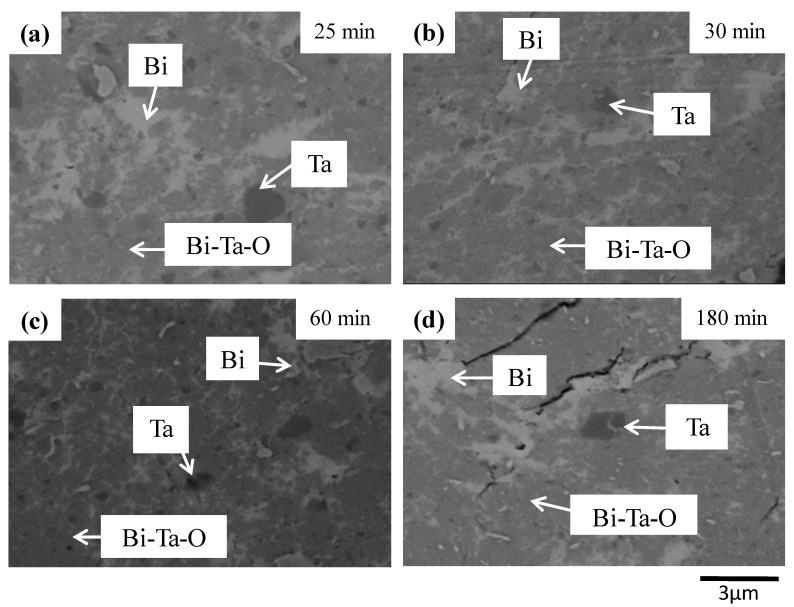
SEM images of (Bi_2_O_3_)_80_Ta_20_ as a function of milling time under Ar-filled glove box conditions: (**a**) 25, (**b**) 30, (**c**) 60, and (**d**) 180 min.

**Figure 4:**
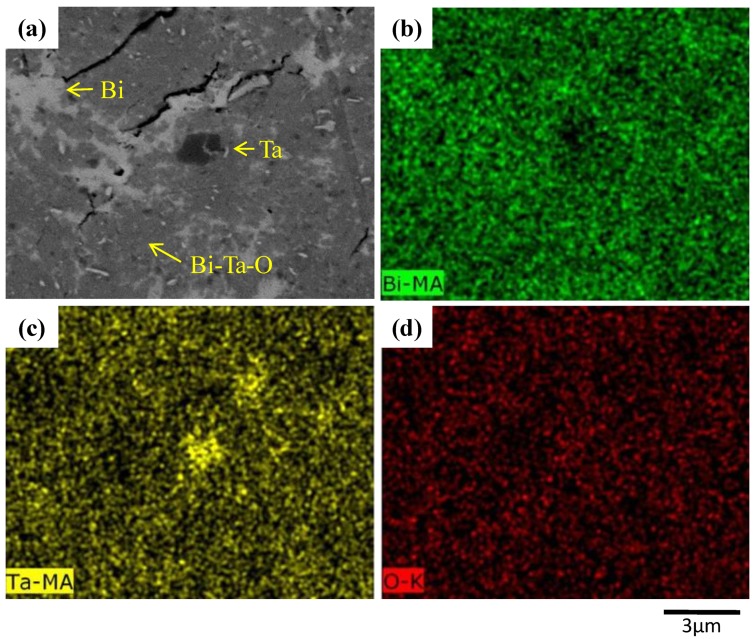
EDX mapping of 3h-as milled (Bi_2_O_3_)_80_Ta_20_ powder. (**a**) SEM image; and mapping of element (**b**) Bi; (**c**) Ta; (**d**) O.

To better demonstrate the structural evolution, the cross-sectional views with higher magnification of selective as-milled powders are shown in Figure 3. Notably, in the SEM photos obtained using backscattered electron images, the larger the atom, the brighter the area. As marked in Figure 3, the brightest area is bismuth, whereas the darkest area is tantalum, with the brightness of Bi-Ta-O being in between. Only the tantalum particle is close to being spherical in shape, whereas the others (Bi and Bi-Ta-O) are irregular. This element distribution was confirmed using energy dispersive X-ray (EDX) mapping. Figure 4 shows the three-hour as-milled powder with bismuth, tantalum, and oxygen mappings, where corresponding elements are uniformly distributed within the examined area, with the exception of some localized regions.

**Figure 5:**
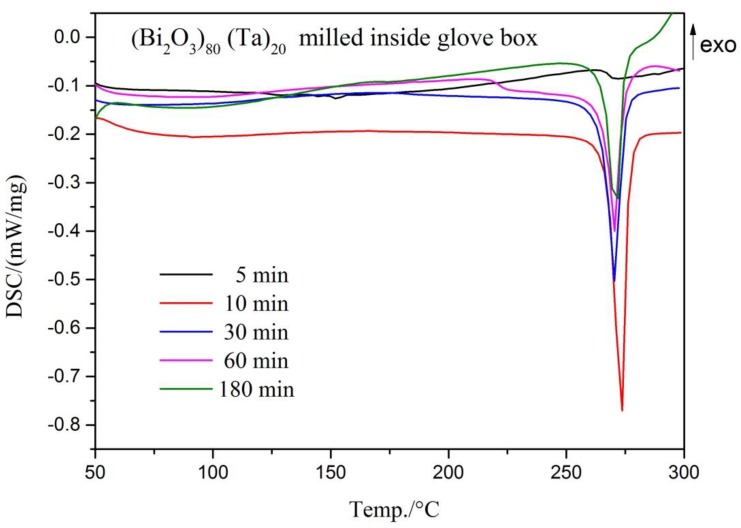
Differential SC curves of (Bi_2_O_3_)_80_Ta_20_ after different milling times under Ar-filled glove box conditions.

Figure 5 shows the DSC curves of (Bi_2_O_3_)_80_(Ta)_20_ powder after different milling times. An endothermic peak around 271.1 °C (the melting point of bismuth) was observed for as-milled (Bi_2_O_3_)_80_(Ta)_20_ powders. The five-minute as-milled powder (black line in Figure 5) did not show a distinct endothermic peak, whereas the 10-min as-milled powder (red line in Figure 5) exhibited the largest endothermic peak at 272.8 °C, which corresponds to the melting of bismuth. The endothermic peak position did not show significant differences with long-term milling (30–180 min). The peak height, however, decreased with increasing milling time. This suggests that the amount of bismuth formed by mechanochemical reaction quickly reached a maximum during the early stage of milling (10 min) and gradually decreased with prolonged milling.

**Figure 6:**
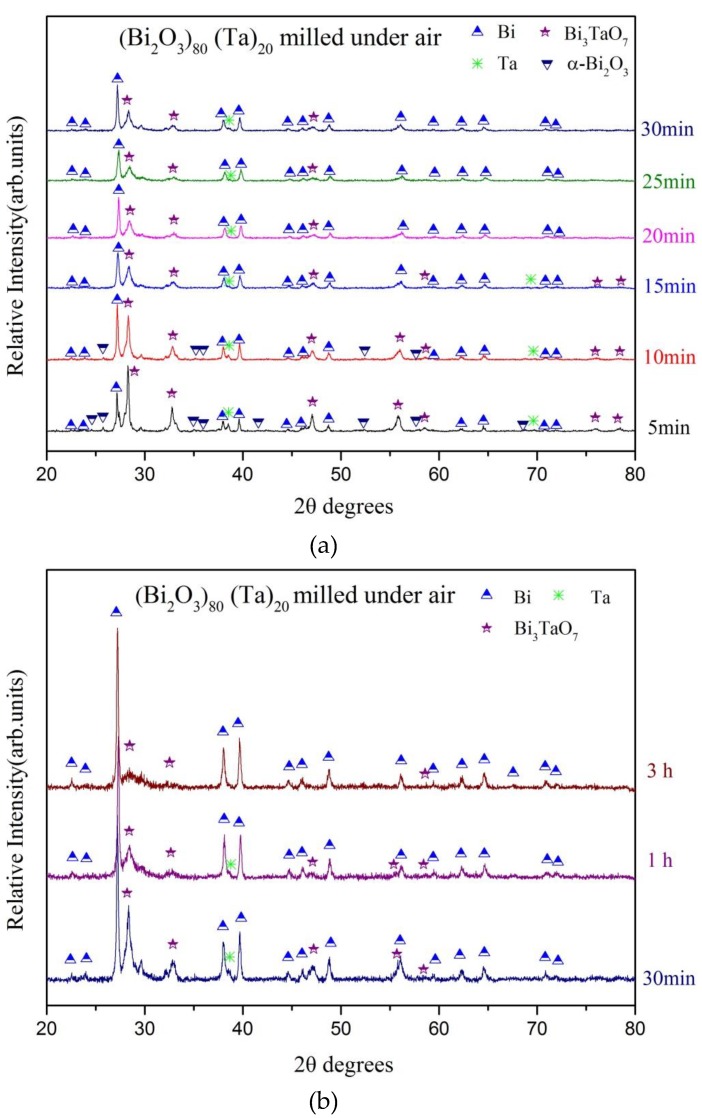
X-ray diffraction patterns of (Bi_2_O_3_)_80_Ta_20_ as a function of milling time under air. (**a**) early stages of milling and (**b**) long-term milling.

The aforementioned high energy ball milling process is performed in a glove box, in which the total O_2_ and H_2_O concentrations are less than 100 ppm. Figure 6 shows the X-ray diffraction patterns of (Bi_2_O_3_)_80_(Ta)_20_ powder produced using high energy ball milling within an ambient environment in which the oxygen concentration is ~20 vol %. As shown in the bottom XRD pattern in Figure 6a, formation of α-Bi_2_O_4_, bismuth and δ-Bi_3_TaO_7_ can be observed after merely 5 min of milling treatment (compared to that of 10 mins. within glove box, Figure 1a). This suggests that the mechanochemical reaction can be accelerated with the aid of oxygen. Similar to that of milling (Bi_2_O_3_)_80_(Ta)_20_ under glove box conditions, no significant differences in peaks’ intensities for α-Bi_2_O_4_ can be observed. Whereas, the diffraction peaks of bismuth reached a maximum after 10 min of milling. Those of the 30-min as-milled powder exhibited a maximum peak height for δ-Bi_3_TaO_7_ phase. The crystalline peaks of α-Bi_2_O_3_ diminished with milling time and became undistinguishable after 15 min of milling (30 min within glove box). However, those of tantalum persisted for up to 1 h of milling and could not be recognized at the end of 3 h milling.

**Figure 7:**
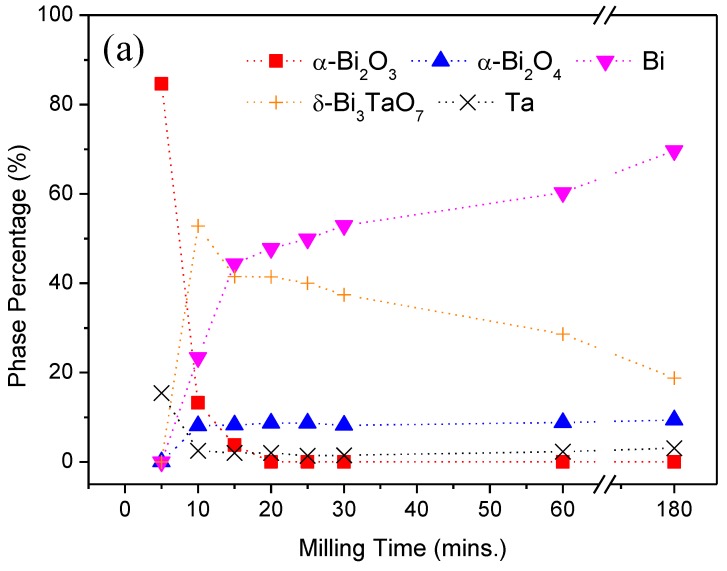

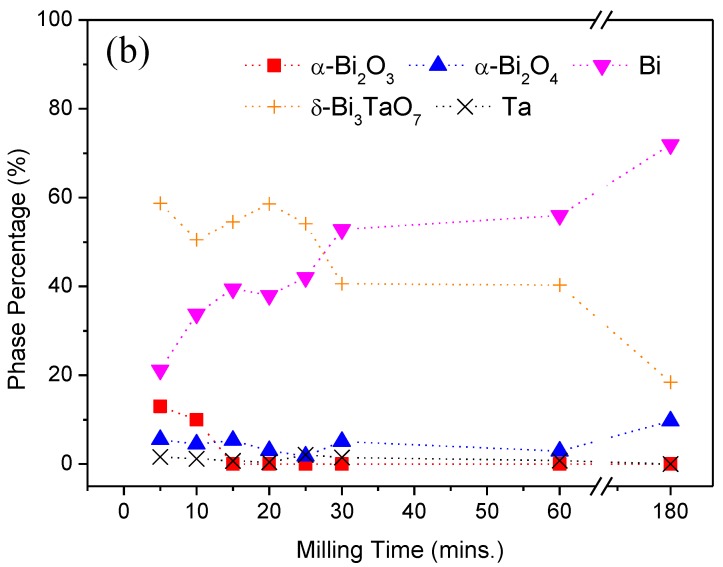
The percentage of individual phase as a function of milling time under (**a**) Ar-filled glove box conditions and (**b**) air.

In order to further reveal the phase transition during ball milling process, the XRD results were analyzed by Rietveld fitting method. Table 1 summarizes the phase transitions and the corresponding phase percentages of (Bi_2_O_3_)_80_(Ta)_20_ powder at various milling stages during high energy ball milling under either glove box or ambient atmosphere conditions. Figure 7 shows the percentage of individual phase as a function of milling time. As shown in Figure 7a for (Bi_2_O_3_)_80_(Ta)_20_ powder milled under Ar-filled glove box conditions, the starting powders, α-Bi_2_O_3_ and Ta, transformed into α-Bi_2_O_4_, bismuth, and δ-Bi_3_TaO_7_ phases. The relative percentage of α-Bi_2_O_4_ remained ~8 to 9% after 10 min of milling and did not show significant difference thereafter. The percentage of Ta was 15.4% after 5 min of milling, decreased to 2.5% at 10 min milling, and fluctuated around 1.4% to 3.1% through the milling process. A gradual increase of bismuth from 23.4% (10 min of milling) to 69.7% (3 h of milling) can be observed. The relative amount of δ-Bi_3_TaO_7_ phase, however, reached a maximum of 52.8% after 10 min of milling and gradually decreased to 18.8% after 3 h of milling treatment. Figure 7b shows the percentage of individual phase for (Bi_2_O_3_)_80_(Ta)_20_ powder milled under ambient atmosphere conditions. Similar behavior can be noticed as those shown in Figure 7a, except for the accelerated mechanochemical reaction after 5 min of milling.

**Figure 8:**
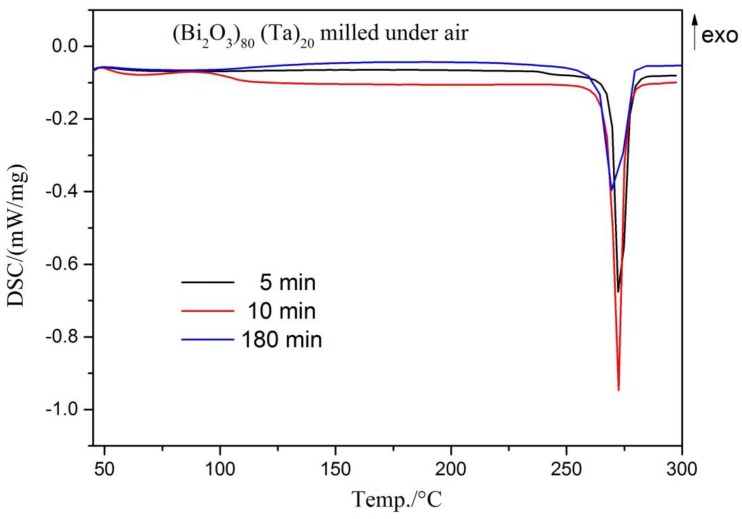
Differential scanning calorimetry (DSC) curves of (Bi_2_O_3_)_80_Ta_20_ for different milling times under air.

**Figure 9:**
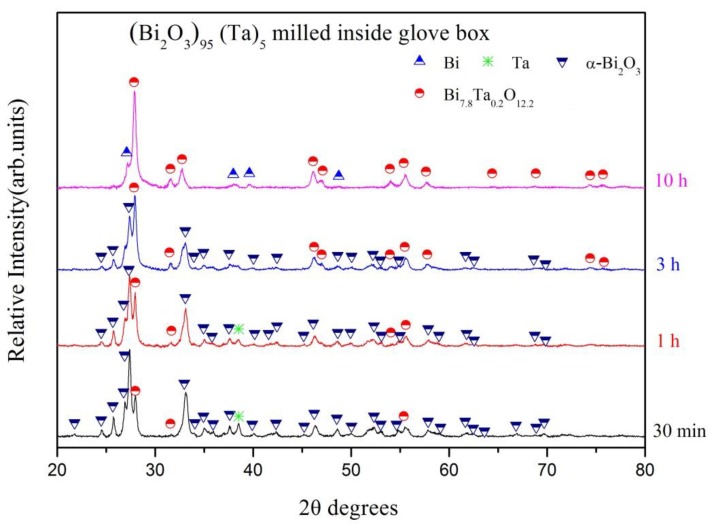
X-ray diffraction patterns of (Bi_2_O_3_)_95_Ta_5_ after different milling times under Ar-filled glove box conditions.

**Figure 10:**
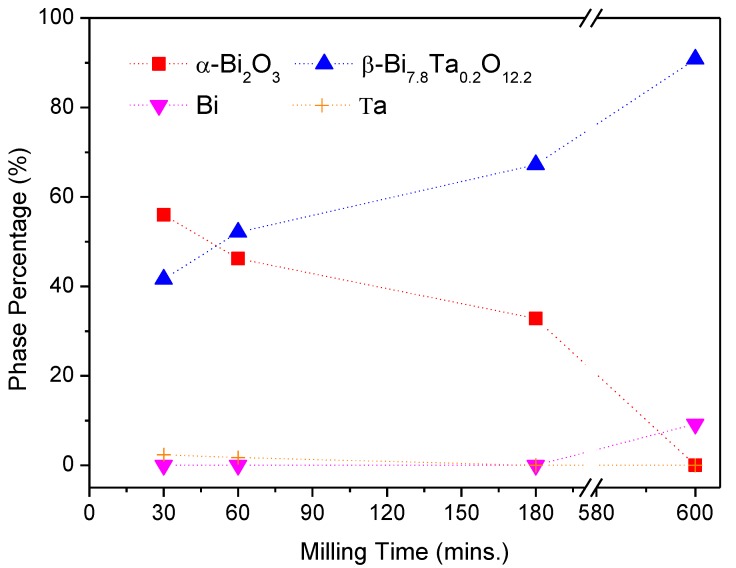
The percentage of individual phase of (Bi_2_O_3_)_95_Ta_5_ after different milling times under Ar-filled glove box conditions.

Though not shown here, cross-sectional examination of as-milled (Bi_2_O_3_)_80_(Ta)_20_ powder under ambient atmosphere showed widely distributed irregular grains at all milling stages and did not reveal significant differences from those milled within the glove box (Figure 2). Figure 8 shows the DSC curves of as-milled powder at various stages. The 5-min as-milled powder exhibited an endothermic peak at 273.1 °C, which is slightly higher than the 271.1 °C melting point of bismuth. This accounts for the existence of bismuth within the 5-mins as-milled powder. After 10-mins of milling, the endothermic peak exhibited a maximum peak height and indicated the reduced bismuth reached a maximum. This confirmed the XRD results discussed in Figure 6. The endothermic peak height decreased with prolonged milling similar to those milled under an Ar-filled glove box environment (Figure 5). DSC examination confirms that the mechanochemical reaction began at a very early stage of milling (5 min) and continued thereafter throughout the ball milling process.

(Bi_2_O_3_)_80_(Ta)_20_ powders milled within either the glove box or ambient environments were used to investigate the effects of oxygen concentration. Additionally, to determine the influence of tantalum addition, the amount of tantalum was decreased to 5 wt % and high-energy ball milling was executed within a glove box. Figure 9 shows the X-ray diffraction patterns of the as-milled (Bi_2_O_3_)_95_(Ta)_5_ powders at various milling stages. With a limited amount of tantalum addition, the superfluous reactant α-Bi_2_O_3_ phase persisted for up to three hours of milling. The crystalline peaks of tantalum were observed after one hour of milling. A new crystalline phase of tetragonal β-Bi_7.8_Ta_0.2_O_12.2_ (ICDD PDF card No. 043-0451) was observed after 30 min of milling. During the three hours of milling, bismuth was not reduced, and tantalum was continuously merged into the α-Bi_2_O_3_ phase, which induced the formation of β-Bi_7.8_Ta_0.2_O_12.2_ phase. When the milling time was increased to 10 h, the crystalline peaks of the starting powders α-Bi_2_O_3_ and Ta disappeared, whereas the peak heights of β-Bi_7.8_Ta_0.2_O_12.2_ phase increased, and diffraction peaks of newly-formed bismuth were observed. Table 2 summarizes the corresponding crystalline phases and relative phase percentages at various milling stages of (Bi_2_O_3_)_95_(Ta)_5_ powders. The relative amounts of individual phases can be observed better in Figure 10, in which α-Bi_2_O_3_ was 56.0% after 30 min of milling, and the amount decreased with increasing milling time and became indistinguishable at the end of milling (10 h). The amount of 30-min as-milled β-Bi_7.8_Ta_0.2_O_12.2_ phase was 41.6%, which gradually increased with increasing milling time and reached 90.8% after 10 h of milling. In addition, the diffraction peaks of Ta disappeared after three hours of milling and the formation of bismuth was observed after 10 h milling.

The starting powder, α-Bi_2_O_3_, exhibits a monoclinic structure that is stable at room temperature. Unlike α-Bi_2_O_3_, δ-Bi_2_O_3_ is fluorite-like cubic structure that is stable at high temperatures (730–825 °C). During phase transformation, two intermediate metastable phases, which are either a tetragonal β phase or a body-centered cubic γ phase, can be observed. In the present study, for the (Bi_2_O_3_)_95_(Ta)_5_ powder mixture milled under glove box conditions, tantalum slowly but continuously reacted with α-Bi_2_O_3_ phase and formed tetragonal β-Bi_7.8_Ta_0.2_O_12.2_ phase. When (Bi_2_O_3_)_80_(Ta)_20_ was milled under glove box conditions, the mechanochemical reaction began after 10 min of high-energy ball milling and formation of fluorite-like δ-Bi_3_TaO_7_ was observed. By milling under ambient environment conditions, the oxygen concentration was higher, allowing the mechanochemical reaction to accelerate, which triggered the reaction within less than five minutes of milling.

## 4. Conclusions

By milling (Bi_2_O_3_)_80_(Ta)_20_ and (Bi_2_O_3_)_95_(Ta)_5_ compositions, high-temperature fluorite-like phases were prepared using a mechanochemical reaction. By milling (Bi_2_O_3_)_95_(Ta)_5_ powder mixtures within an Ar-filled glove box, tantalum gradually reacted with α-Bi_2_O_3_ phase and formed β-Bi_7.8_Ta_0.2_O_12.2_ phase after 10 min of milling. By increasing tantalum addition to 20 wt %, superfluous Ta can be mechanochemically reacted with α-Bi_2_O_3_ to form δ-Bi_3_TaO_7_ and bismuth phases after 10 min of ball milling. The mechanochemical reaction can be shortened to less than five minutes by increasing oxygen concentration (i.e., milling (Bi_2_O_3_)_80_(Ta)_20_ under ambient atmosphere conditions), which resulted in the formation of a high temperature δ-Bi_3_TaO_7_ phase.

## Figures and Tables

**Table 1:** Crystalline phases of (Bi_2_O_3_)_80_Ta_20_ as a function of milling time under different oxygen environments.

Milling Condition	Milling Time	Crystalline Phases
Inside Ar-filled glove box	5 min	α-Bi_2_O_3_ (84.6%) * + Ta (15.4%)
	10 min	α-Bi_2_O_4_ (8.1%) + α-Bi_2_O_3_ (13.2%) + Bi (23.4%) + δ-Bi_3_TaO_7_ (52.8%) + Ta (2.5%)
	15 min	α-Bi_2_O_4_ (8.3%) + α-Bi_2_O_3_ (3.8%) + Bi (44.4%) + δ-Bi_3_TaO_7_ (41.5%) + Ta (2.0%)
	20 min	α-Bi_2_O_4_ (8.7%) + Bi (47.8%) + δ-Bi_3_TaO_7_ (41.4%) + Ta (2.0%)
	25 min	α-Bi_2_O_4_ (8.7%) + Bi (49.9%) + δ-Bi_3_TaO_7_ (40.0%) + Ta (1.4%)
	30 min	α-Bi_2_O_4_ (8.2%) + Bi (52.9%) + δ-Bi_3_TaO_7_ (37.4%) + Ta (1.5%)
	1 h	α-Bi_2_O_4_ (8.8%) + Bi (60.3%) + δ-Bi_3_TaO_7_ (28.6%) + Ta (2.3%)
	3 h	α-Bi_2_O_4_ (9.4%) + Bi (69.7%) + δ-Bi_3_TaO_7_ (18.8%) + Ta (3.1%)
Under air	5 min	α-Bi_2_O_4_ (5.5%) + α-Bi_2_O_3_ (13.0%) + Bi (21.2%) + δ-Bi_3_TaO_7_ (58.7%) + Ta (1.6%)
	10 min	α-Bi_2_O_4_ (4.5%) + α-Bi_2_O_3_ (10.0%) +Bi (33.8%) + δ-Bi_3_TaO_7_ (50.5%) + Ta (1.2%)
	15 min	α-Bi_2_O_4_ (5.3%) + Bi (39.5%) + δ-Bi_3_TaO_7_ (54.5%) + Ta (0.7%)
	20 min	α-Bi_2_O_4_ (3.0%) + Bi (38.0%) + δ-Bi_3_TaO_7_ (58.6%) + Ta (0.4%)
	25 min	α-Bi_2_O_4_ (1.8%) + Bi (42.0%) + δ-Bi_3_TaO_7_ (54.1%) + Ta (2.1%)
	30 min	α-Bi_2_O_4_ (5.1%) + Bi (52.8%) + δ-Bi_3_TaO_7_ (40.6%) + Ta (1.5%)
	1 h	α-Bi_2_O_4_ (2.9%) + Bi (56.0%) + δ-Bi_3_TaO_7_ (40.3%) + Ta (0.8%)
	3 h	α-Bi_2_O_4_ (2.7%) + Bi (71.9%) + δ-Bi_3_TaO_7_ (25.4%)

Note: *: the percentage of individual phase is given in the bracket

**Table 2:** Crystalline phases of (Bi_2_O_3_)_95_Ta_5_ as a function of milling time under Ar-filled glove box conditions.

Milling Condition	Milling Time	Crystalline Phases
Inside Ar-filled glove box	30 min	α-Bi_2_O_3_ (56.0%) * + Ta (2.4%) + β-Bi_7.8_Ta_0.2_O_12.2_ (41.6%)
	1 h	α-Bi_2_O_3_ (46.2%) + Ta (1.7%) + β-Bi_7.8_Ta_0.2_O_12.2_ (52.1%)
	3 h	α-Bi_2_O_3_ (32.8%) + β-Bi_7.8_Ta_0.2_O_12.2_ (67.2%)
	10 h	β-Bi_7.8_Ta_0.2_O_12.2_ (90.8%) + Bi (9.2%)

Note: * the percentage of the individual phase is provided in the bracket.
